# Hardware/Software Data Acquisition System for Real Time Cell Temperature Monitoring in Air-Cooled Polymer Electrolyte Fuel Cells

**DOI:** 10.3390/s17071600

**Published:** 2017-07-09

**Authors:** Francisca Segura, Veronica Bartolucci, José Manuel Andújar

**Affiliations:** 1 Department of Electronic, Computer Science and Automatic Engineering, University of Huelva, Engineering High School, Crta. Huelva- Palos de la Fra, 21919 Palos de la Fra, Huelva, Spain; andujar@diesia.uhu.es; 2 Dipartimento di Ingegneria dell’Informazione, Universita’ Politecnica delle Marche, Via Brecce Bianche, 60131 Ancona, Italy; s1080408@studenti.univpm.it

**Keywords:** data acquisition, polymer electrolyte fuel cell, real time cell temperature monitoring, virtual instrument, Arduino

## Abstract

This work presents a hardware/software data acquisition system developed for monitoring the temperature in real time of the cells in Air-Cooled Polymer Electrolyte Fuel Cells (AC-PEFC). These fuel cells are of great interest because they can carry out, in a single operation, the processes of oxidation and refrigeration. This allows reduction of weight, volume, cost and complexity of the control system in the AC-PEFC. In this type of PEFC (and in general in any PEFC), the reliable monitoring of temperature along the entire surface of the stack is fundamental, since a suitable temperature and a regular distribution thereof, are key for a better performance of the stack and a longer lifetime under the best operating conditions. The developed data acquisition (DAQ) system can perform non-intrusive temperature measurements of each individual cell of an AC-PEFC stack of any power (from watts to kilowatts). The stack power is related to the temperature gradient; i.e., a higher power corresponds to a higher stack surface, and consequently higher temperature difference between the coldest and the hottest point. The developed DAQ system has been implemented with the low-cost open-source platform Arduino, and it is completed with a modular virtual instrument that has been developed using NI LabVIEW. Temperature vs time evolution of all the cells of an AC-PEFC both together and individually can be registered and supervised. The paper explains comprehensively the developed DAQ system together with experimental results that demonstrate the suitability of the system.

## 1. Introduction

The problem this paper attempts to resolve is (at least the proposal by the authors) the monitoring in a reliable way of the cell temperature across the entire stack surface of a Polymer Electrolyte Fuel Cell (PEFC). Ideally, excess renewable energy generated during times of plenty can be stored for later use during periods when sufficient renewable electricity is not available. Energy storage in the form of hydrogen is one such possibility: excess electricity is fed into an electrolyzer to split water into its constituent parts, oxygen and hydrogen. The produced hydrogen is then used in fuel cells to supply electricity when it is needed (absence or shortage of renewable energy sources like solar and/or wind), releasing the stored energy back to the grid (standalone or not) [1].

Within the range of fuel cells, PEFCs are a promising technology to produce electricity from hydrogen for stationary power generation due to their operational strengths, such as high power density, low operating temperature, low corrosion, quiet operation, stack design simplification, relatively quick startup and shutdown, and especially their zero emission capability [2].

PEFC technology is becoming more and more important because it is suitable for a wide range of applications, including portable, stationary and automotive power delivery [3] and lately it is being used more in backup systems for emergency situations (e.g., earthquakes, terrorist attacks) [4]. Configuration or hybridization of generation systems around the PEFC can be miscellaneous [5,6] as well as its control modes [7].

The two key parts of a PEFC are the stack (assembly of stacked cells) and the Balance of Plant (BoP). In summary, the BoP is integrated by five subsystems: Oxidant, Fuel, Cooling, Electrical and Control. The role of these subsystems is to supply reactants (oxygen and hydrogen at the appropriate flow and pressure for electrochemical reaction), remove the heat generated in the stack, maintain it at the temperature recommended by the manufacturer, eliminate the water produced, and connect the stack to an electric load and process information from sensors to control the actuators. The electrochemical reaction, which generates electricity from hydrogen and air, takes place in the stack. The BoP’s function is to make the stack work properly [8,9,10].

While a PEFC is operating, the stack temperature changes with time, and it depends on the load demand. The temperature has influence on the electrochemical reaction rate and on water and reactant transport. At the individual cell level, low temperatures may produce membrane flooding and because of that, operating problems might appear due to membrane resistivity variation. Otherwise, high temperatures might produce membrane thermal stress and cathode catalyst inactivity, resulting in membrane degradation [11,12]. Consequently, a good regulation of temperature in the stack is key, at the cell level if possible, which requires temperature measurements with a degree precision.

Thus, a cell’s temperature monitoring system will provide information about the state of each cell and this will allow the user to prevent or avoid cell failures. It is common to find commercial stacks that the manufacturer supplies with a temperature sensor, but a single temperature measurement does not inform about what happens in each cell [13].

The techniques dedicated to monitor the state of the fuel cells are known as Prognostics and Health Management (PHM) methods [14] and they consist of collecting data during normal operation and treating them subsequently to identify the origin of any malfunction. On the basis of the functionality of PHM, these methods can be used not only to determine faults in the system, but they are also part of the control system; indeed the control system will make decisions on the basis of the information supplied by PHM. As stated by Calderón et al. [15] most PHM methods utilize cell voltage as main parameter for fault detection, but aside from voltage, cell temperature is another variable of great importance to be used by PHM methods (this is the main novelty of this paper). For example, a cell working at temperatures above the range recommended by the manufacturer is a clear evidence of defective functioning.

**Figure 1:**
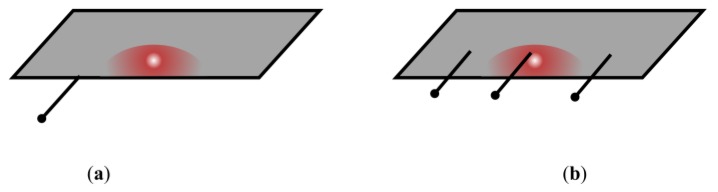
Comparison between cell voltage-based PHM method (**a**) and cell temperature-based PHM method (**b**).

Even more, comparing cell voltage-based PHM methods versus cell temperature-based PHM methods; cell temperature-based methods can give a clue about which side of the cell is more damaged. This can be achieved by placing multiple temperature sensors on different parts of the cell (for example on the right, on the middle and on the left, see Figure 1). The side where the cell is damaged will present a higher temperature than the rest of the surface. In contrast, if the prognostics method was based on voltage sensing, this fault would not be as easily detectable.

Several factors affect the durability and efficiency of PEFCs: ageing, component degradation, impurities, air composition, materials, catalyst, water management, temperature, membrane humidity, heterogeneous distributions of operating conditions inside the cell, and cell voltage reversal [16].

In this paper, a hardware/software data acquisition system for real time monitoring of cells’ temperature in Air-Cooled Polymer Electrolyte Fuel Cells (AC-PEFC) is developed. The temperatures of all the cells in an AC-PEFC stack, both together and individually, are recorded and monitored. For the development of the data acquisition (DAQ) system, we can choose between different software tools like Wonderware InTouch [17] (from the multinational enterprise Schneider Electric, Rueil-Malmaison, Hauts-de-Seine, France), IntellutioniFIX [18] (by the multinational General Electric, Boston, Massachusetts, USA), Siemens WinCC [19] (Siemens, Berlin, German), LabVIEW [20] (National Instruments, Austin, Texas, USA), as proprietary options. Among open-source options, we can find RapidSCADA [21] (RapidSCADA, Moscow, Russian Federation), OpenSCADA [22] (IBH Systems GmbH, Pfaffenhofen an der Glonn, Bavaria, Germany), IndigoSCADA [23] (enscada, Busto Arsizio, Lombardy, Italy). In this case, we have used NI Laboratory Virtual Instrumentation Engineering Workbench (LabVIEW) because of its widespread use around the world and the fact it supports several applications [24]. For example, Pany et al. [25] developed NI LabVIEW programs to reduce the stress on an electric drive fed by a fuel cell based hybrid source. Barbouche et al. applied [26] a monitoring system based on NI LabVIEW to a fuel cell test station to enable a mass flow controller and the solenoid valves control. Additionally, Segura [27] and Andújar [28], presented respectively a SCADA system implemented under NI LabVIEW for simulation and real-time monitoring of a modular PEFC system.

On the other hand, in relation to hardware implementation of the DAQ system, the authors have opted for an Arduino platform [29] rather than other alternatives like RaspberryPi [30], Intel Edison [31] and openDAQ [32]. In this case, the only necessity is for a microcontroller able to receive signals provided by sensors and transmit them to a monitoring system. The Arduino platform is a better and cheaper option for this case as there is no need for any Operating System (OS) functionality or software applications to run, since all that needs to be done is write the code.

Regarding the scientific works focused on developing thermal sensors for fuel cells, Lee et al. [33] presented a flexible three-in-one microsensor to measure temperature, voltage and current in a lithium battery cell. Later, they succeeded in developing a device comprising five microsensors to measure in-situ temperature, voltage, pressure, fuel flow and current, but in this case for high temperature fuel cells (HT-FCs) [34]. The utility of this five-in-one sensor is justified because lack of consistency in any of these variables will affect the fuel cell performance and consequently its lifetime.

The rest of the most recent papers also work under the same concept of integrated sensors. Zheng et al. designed in [35] a wireless temperature and humidity sensor for microbial fuel cells. In this case, the fuel cell supplies the sensor, but the stack voltage depends on the Power Management System (PMS) that the authors applied. Then, to guarantee a continuous electrical supply to the sensor and to avoid its intermittency both in the supply and in its functioning, it is necessary to define a correct and reliable PMS. The concern that intermittency presents is solved by Kuo et al. [36] where a similar (humidity and temperature) sensor is used but with the added requirement of an auxiliary power supply. In the following sections, the hardware, software and procedure to implement the developed DAQ system and its experimental verification are described. Regarding commercial systems, that what has been found [37] present poor resolution, more wiring requirement and it need additional power supply.

## 2. Materials and Methods

The testing bench for this study (an AC-PEFC system) has been designed and built around the air-cooled FCgen-1200ACS stack model from Ballard^®^ (Burnaby, BC, Canada) [38]. The stack is made up of 80 cells and it can supply 80 V without load and reach up to 3.4 kW at 75 A and 45.3 V. At nominal current, the optimal operating temperature and pressure values are 67 °C and 1.36 bar, respectively.

The AC-PEFC study integrates the oxidant and cooling subsystems into one, and the oxidant/cooling agent can be the surrounding air. That is, it is not necessary to add an additional water circuit to cool the system because the same air managed by the oxidant subsystem is responsible for cooling the stack. Thanks to this feature, the fuel cell features reduced weight, volume, cost and control complexity.

**Figure 2:**
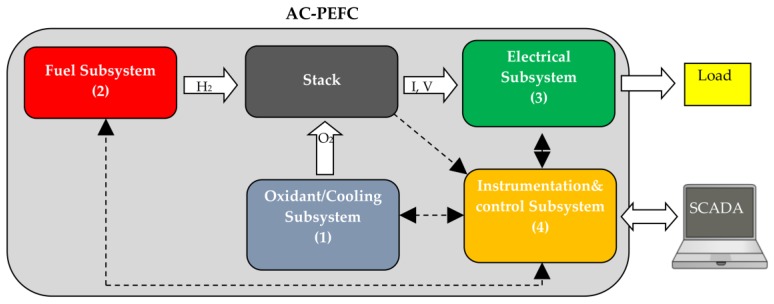
AC-PEFC test bench and SCADA for its monitoring and control.

A fuel cell is an assembly integrated by a stack and several subsystems that make up the BoP. In the particular case of AC-PEFCs, the BoP can be divided into the following subsystems (see Figure 2): (1) Oxidant/Cooling subsystem: this crucial subsystem supplies air/oxygen (oxidant) at the appropriate conditions for the oxidant reaction, and it removes the heat (cooling) produced in the stack and keeps it at the right temperature; (2) Fuel subsystem: it supplies hydrogen at the appropriate conditions for the reduction reaction; (3) Electrical subsystem: it connects the stack to electric load; and (4) Instrumentation & Control subsystem: it processes sensors information to control actuators.

The test bench management is carried out by a Supervisory Control and Data Acquisition (SCADA) system (Figure 2) also developed by the authors. It is hosted on a PC and it is provided with a data network connection for remote management, as well as a graphical user interface. It can also manage others peripheral devices (PIDs, sensors, actuators and so on) with the Instrumentation & Control subsystem. This means that, with the SCADA, there can be absolute control of the test bench and all its information can be processed as well.

**Figure 3:**
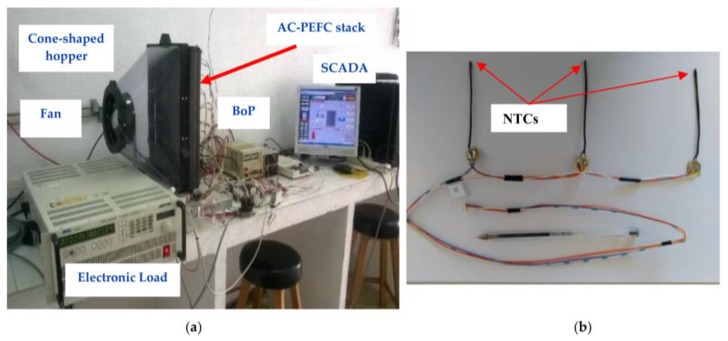
(**a**) AC-PEFC test bench. (**b**) Detail of a group three NTCs for three measurement points on a cell; normally left, center and right.

This test bench, which has been designed to put in work AC-PEFCs, has been fully developed by the authors of this work and it is shown in Figure 3a.

To measure the cell temperature, we have used Negative Temperature Coefficient (NTC) sensors, also called thermistors, i.e., thermally sensitive resistors. Figure 3b shows a set of three NTCs wired and arranged to be placed in a cell. As we can see, each sensor is placed on a fixed-length rod that is inserted in a cathode flow channel; the stack is based on an open-cathode design.

For implementing part of the hardware of the developed DAQ system, we have used an Arduino Micro ATmega32u4 Microcontroller and for developing the SCADA software, we have used LabVIEW. Please see Section 2.1 for a detailed description of the DAQ system.

Finally, the experimentation has been conducted using the FCTESTNET/FCTESQA PEFC power stack performance testing procedure [39]. It consists of increasing the load demand gradually from null to full load, and once the maximum operating point has been achieved, the load demand will descend in the same step amplitude that was used to increase the load. The aim of the procedure is to subject the AC-PEFC stack to a changing load profile. Then, the stack temperature, and consequently the cell’s temperature, will rise first, and after it will descend according to the load demand. The developed DAQ system will allow us to have the cells’ temperature measurements and with them, locate any damaged cell or identify some malfunctioning of the stack.

### 2.1. Developed Data Acquisition System

#### 2.1.1. Hardware Development

**Figure 4:**
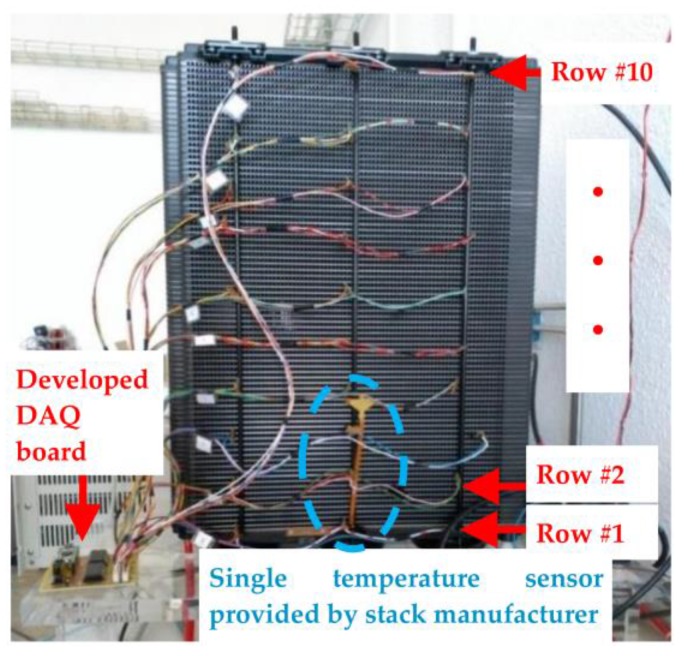
Stack under study with all the temperature sensors connected.

The measurement hardware is made from an array of NTC sensors. This array consists of a matrix of 10 rows and three columns (left, center and right of the cell), i.e., 30 NTC sensors. As we have mentioned above, each sensor is placed on a fixed-length rod, Figure 3b, which is inserted (Figure 4) on a cathode flow channel (the stack is based on an open-cathode design). This implementation assures that all the NTCs are in the same depth in the cell for all the 30 sensors. Additionally, the NTC sensor placement in the cells does not affect the performance of the cell because both the size of the sensor and the wiring are small enough to impede air from entering the cathode channel. Additionally, to perform a measurement point distribution that can provide a complete stack map, we have decided to place 10 sensing rows, leaving eight free cells between each sensing row (see Figure 4). Of course, for each particular application, the array of sensors should match the size of the stack (length and width). The manufacturer provides the stack equipped with only one temperature sensor (also a NTC), which is indicated in Figure 4. This will allow us to compare the experimental results obtained from the developed DAQ system with regard to the given temperature collected from the sensor that the stack incorporates. Precisely for this reason, we have decided to use the same NTC as the manufacturer, specifically the NTC NB20N50104KBA (please see Table 1).

The NTC thermistors (*T*) are ordered in the stack surface by rows and columns (*T_i,j_*), and they are put on the cathode side of the bipolar plates of the stack. Each sensing row is made up of three NTCs (see Figure 3b) which are placed on the borders (left and right) and on the middle of the cell (*T_i_*_,1_, *T_i_*_,2_ and *T_i_*_,3_). This allows sensing of the temperature of the entire surface because of the 3-point measurements for each cell. Additionally, each row is repeated ten times along the stack (from *T*_1,*j*_ row up to *T*_80,*j*_ row), leaving eight cells (the stack used it this work has 80 cells) between each pair of sensing rows (Figure 4). Of course, this distribution can be adapted to any other structural stack design.

Because of the design, we must process 30 analog signals. To do this with a single conditioning circuit, we have decided to multiplex these signals. The thermal inertia [40] at the measurement points is much slower than the acquisition time of a multiplexer; therefore, it is not necessary to acquire the 30 temperature measurements at exactly the same time.

**Figure 5:**
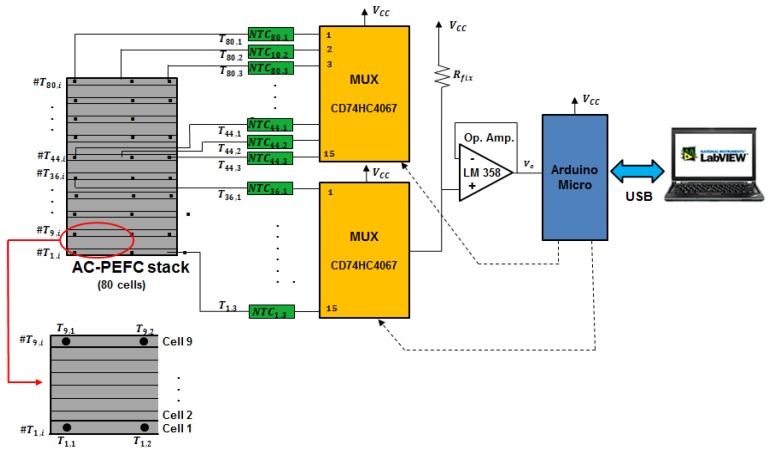
DAQ system architecture.

Following this reasoning, we have used two 16-channels analog multiplexers to multiplex the 30 temperature measurement signals. All the multiplexed signals go to the same conditioning circuit built with a general-purpose operational amplifier (Op. Amp., see Figure 5). This can be a good solution, since as the sensors are the same; the electrical variable to be processed is also the same, as well as its range of values.

According to manufacturer data [41], the maximum temperature variation is given by a cell located in the middle of the stack (the hottest point, cell #40) and these placed on the upper (cell #80) and on the lower border (cell #1). This difference between the hottest and coldest points can rise up to 8 °C, so this corresponds to a maximum temperature variation of 0.2 °C/cell. In our case, as we have commented above, there are 10 sensing rows distributed along the whole stack and this corresponds to leaving eight free cells between each sensing row. This will correspond to a maximum variation of 8 cells × 0.2 °C/cell = 1.6 °C. Then, the developed DAQ system for cell temperature monitoring will give us enough temperature information about the cell temperature distribution. Additionally, distributing the sensors rows along the stack skipping the same number of cells between each consecutive sensing row, will give a real idea about the whole temperature distribution. If the user would like to have measurements in all the cells of the stack under study, 80 × 3 = 240 NTC sensors would be needed and therefore 240/16 = 15 analog multiplexers. However the goal of the paper and the developed prototype is to demonstrate the feasibility of the proposal, for which we have limited the number of analog inputs to 32 (two multiplexers of 16 inputs each one), with which we can cover 10 cells with three measurements each one (30 NTCs). However, the scalability of the design allows the number of inputs to be as many as the user desires. For example, by maintaining the same scheme as in Figure 5 and placing an additional multiplexer governed by Arduino, we could increase the number of DAQ inputs to 48 (16 × 3), and so on.

The conditioning circuit adapts the amplitude of the signal to the Arduino input. The Arduino microcontroller is responsible for governing the opening of multiplexer inputs, electrical supply to all electronics, convert the analog temperature signals into digital words and communicate the hardware with the modular Virtual Instrument (VI) (please see Appendix A for the Arduino script).

From the Arduino, all the temperature data are transmitted in digital format (by a USB port) to the modular VI. To simplify the DAQ system and make it very flexible and portable, its same USB port serves as the Arduino power supply and from it to the rest of the electronics.

Table 1 summarizes the main characteristics of the devices used in the developed DAQ system. As it has been said above, the DAQ system has been designed with the aim it does not need an addtional power supply source beyond the one provided from the USB port integrated in it. Therefore, the devices have been chosen to be supplied by 5 V.

Below are provided all the calculations necessary for the practical implementation of the DAQ system architecture.

The voltage drop measured in the voltage divider and presented to the Arduino input (see Figure 5) follows Equation (1):

(1) $$V_{o}\;=\;V_{CC}\frac{R_{NTC\_ij\;}\;+\;R_{on}}{R_{fix}\;+\;\left(R_{NTC\_ij}+\;R_{on}\right)}$$

where:

$V_{CC}$ is the power supply source voltage (5 V).
$R_{NTC\_ij}$ is the the NTC resistance on the part *j* of the cell *i*.
$R_{on}$ is the multiplexer resistance in conduction.
$R_{fix}$ is the fixed resistance used in the voltage divider.

From (1), we can obtain the value of the NTC resistance:

(2) $$R_{NTC\_ij}=\frac{R_{fix}\;V_{o}}{V_{CC}-V_{o}}-R_{on}$$

On the other hand, from the NTC manufacturer data sheets, particularized for the NTC # *ij*, is expressed by Equation (3):

(3) $$R_{NTC_{ij}}=R_{0}\;e^{B\left(\frac{1}{T_{ij}}\;-\;\frac{1}{T_{0}}\right)}$$

where: $R_{0}$ is 100 Ω at $T_{0}$ = 25 °C. B is 4160 K.

From Equation (3), we can obtain the temperature value (in Kelvins) on the part *j* of the cell *i*:

(4) $$T_{ij}\left(K\right)=\frac{B}{\frac{B}{T_{0}}+ln\left(\frac{R_{NTC\_ij}}{R_{0}}\right)}$$

Substituting now in this expression the value of $R_{NTC\_ij}$ from Equation (3), we can find the sensed temperature in Celsius degrees (°C):

(5) $$T_{ij}\left({}^{\circ}C\right)=\frac{B}{\frac{B}{T_{0}}+ln\left(\frac{R_{fix}V_{o}}{R_{0}\left(V_{CC}\;-\;V_{o}\right)}\;-\;\frac{\;R_{on}}{R_{0}}\right)}\;-273$$

Equation (5) allows us to know the temperature of the NTC #*ij*, in terms of known parameters (B, $T_{o}$, $R_{on}$ and $V_{CC}$) and the measurable variable $V_{0}$.

**Figure 6:**
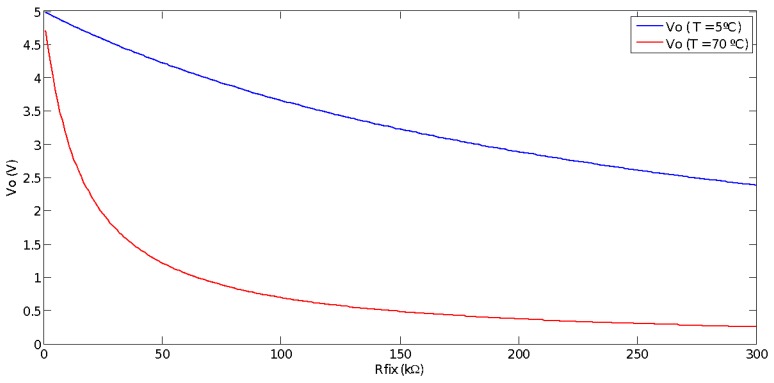
Dependence between measured voltage $V_{o}$ and *R_fix_* at different stack operating temperatures.

Arriving at this point, the only parameter whose value has not been yet justified is $R_{fix}$. Its value has been selected on basis of the following criterion: according to expression (1), measurable voltage, $V_{o}$, depends on the NTC resistance, $R_{NTC\_ij},$ and in turn the NTC resistance depends on the stack operating temperature which, in this application, can vary from 5 °C–70 °C. Then, to choose the most suitable $R_{fix},$ we have used an algorithm which represents $V_{o}$ versus $R_{fix}\;$(varying $R_{fix}$ from 0–300 KΩ), and taking into account (from (3)) the minimum and maximum value of $R_{NTC},$ i.e., $R_{NTC\_min}=15.18$ KΩ (T = 70 °C) and $R_{NTC\_max}\;=272.5$ KΩ (T = 5 °C) (Figure 6).

**Figure 7:**
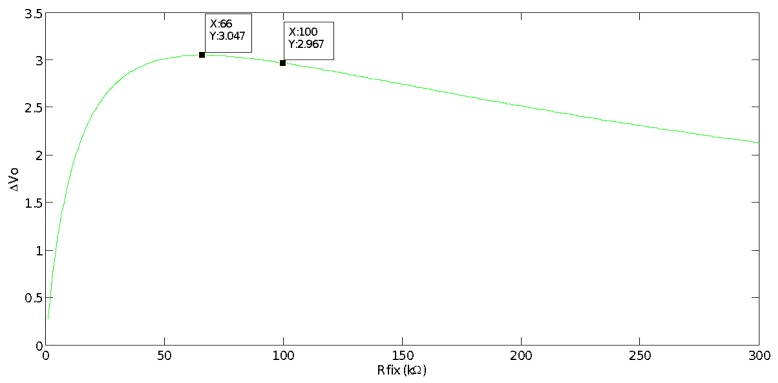
Difference between maximum and minimum voltage ($\Delta V_{o}$), at 5 °C and 70 °C respectively, versus *R_fix_*.

Figure 7 presents the difference between the maximum and minimum value $(\Delta V_{o}=\;V_{omax}-V_{omin}$) of the voltage measured on the voltage divider (see Figure 5).

**Figure 8:**
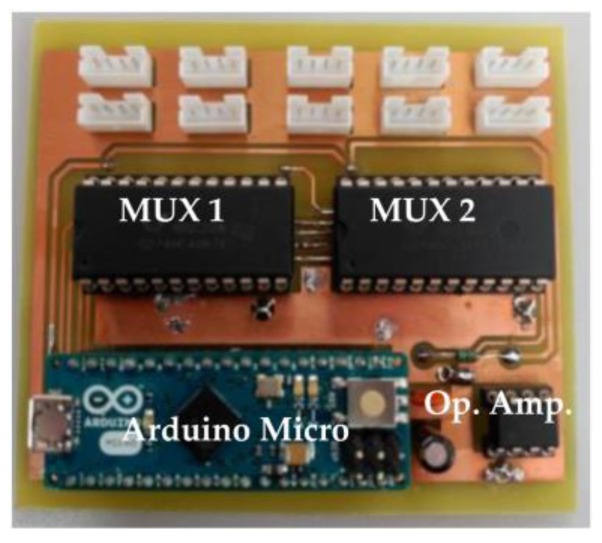
DAQ system: Detail of the final PCB board.

We can observe that by selecting $R_{fix}$ = 66 kΩ, the voltage divider provides the maximum voltage difference between the two extreme operating temperature values. The greater the difference between maximum and minimum voltage the better the measured temperature precision. However, considering commercial resistance values, we have chosen $R_{fix}$ = 100 kΩ. The maximum voltage difference measured with $R_{fix}$ = 66 kΩ is $\Delta V_{o}=\;$3.047 V, while with $R_{fix}$ = 100 kΩ, it is $\Delta V_{o}$ = 2.976 V, so barely 0.08 V are lost and it is not worth building a resistance of 66 kΩ by using a potentiometer in series with a resistance. Finally, Figure 8 shows the DAQ system hardware, i.e., the physical implementation of the Figure 5 scheme.

#### 2.1.2. Software Development

**Figure 9:**
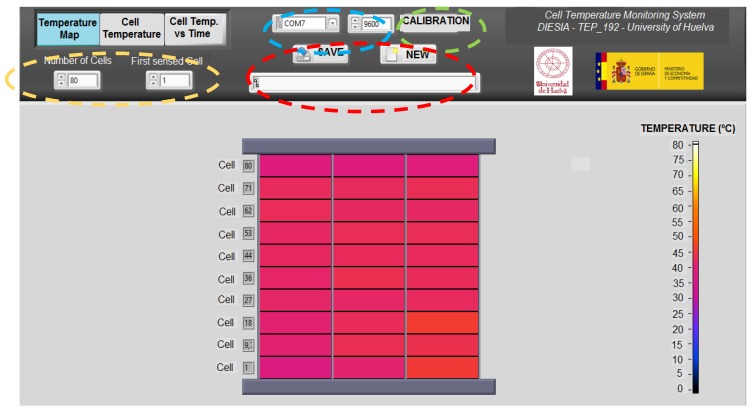
Stack temperature distribution and common panel specifications (stack parameters, data saving settings, communication settings, calibration).

**Figure 10:**
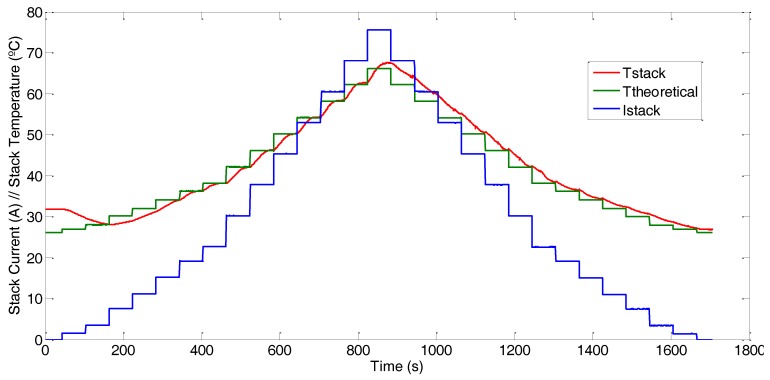
Load profile (**blue**) and stack temperature: real (**red**) and theoretical response (**green**).

**Figure 11:**
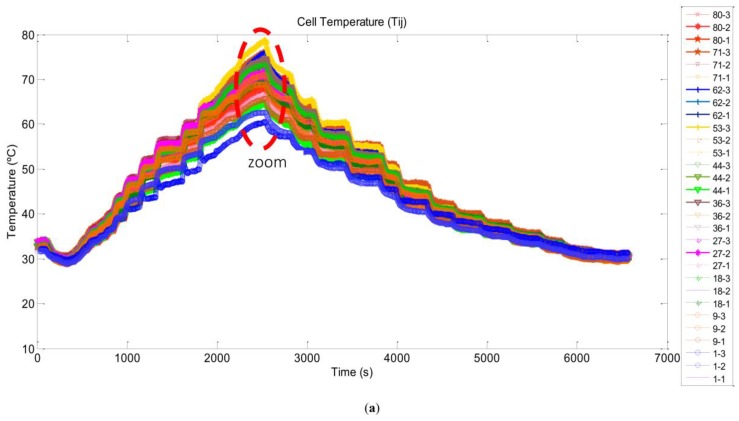

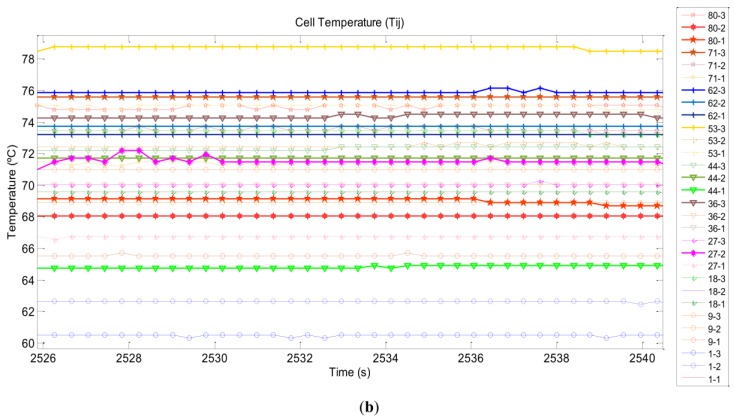
Cells temperature response to load profile measured by the developed DAQ system (**a**) and zoom at peak current (**b**).

For real-time monitoring of the temperature in the AC-PEFC stack, a modular VI has been developed with NI LabVIEW. Communication with the Arduino is programmed directly with NI LabVIEW using the Arduino toolkit. Basic Arduino Sketch for interfacing with LabVIEW provides support for this task. The developed modular VI consists on a common panel (upper side on Figure 9, Figure 10 and Figure 11), where we can find:

∙ An indicator showing the date and time elapsed since the program was started.
∙ A box from which we can chose the port and the speed of the serial communication.
∙ A button to make the calibration.
∙ A box from which we can choose the destination of the data to be saved and the beginning of the data storage.
∙ Two boxes where we can select the number of cells of the stack and the cell from which the user desires to begin the monitoring. Once these two parameters are defined, the modular VI will automatically make a homogeneous distribution, to distribute the group of NTCs in such a way as to have the most equitable distance between them.

Apart from this common panel, the modular VI includes three windows that help to monitor the stack temperature distribution. Then, as a sample of this VI, Figure 9 is included to show the first window that represents the temperature map. This window illustrates the AC-PEFC stack is differentiated by sensing rows, and each sensing row is divided into its three parts (left, middle and right). Additionally, a graded color bar helps to identify the associated temperature at each sensed point. In this way, during an experimental test, we can see how the stack gradually changes its color, which means that the stack temperature is progressively changing. This allows the user to obtain at a glance a very illustrative demonstration of the strengths and weaknesses of the designed oxidant/coolant subsystem, the BoP implementation or information about any damaged cell.

Apart from this, the modular VI offers the possibility to have a temperature chart of the cells where it is represented the temperature in each zone of the stack (sensed by each NTC). Then, we can visualize and compare all the cells temperature values at the same time. Additionally, a representation of cells’ temperature evolution over time is also possible (readers may contact corresponding author for more details).

To conclude this section, Table 2 summarizes the main technical specifications of the presented DAQ system for cell temperature monitoring.

## 3. Experimental Results

The experiment has been conducted using the test bench shown in Figure 3a. The aim is to subject the AC-PEFC stack to a changing load profile, Figure 10 (blue line), so the stack temperature will rise according to load demand (i.e., according with the stack current, $I_{stack}$). For the FC1020-ACS stack from Ballard^®^ used in the test bench, the manufacturer establishes a dependence of theoretical stack temperature, $T_{theoretical}$ (°C), regarding stack current, $I_{stack}$, according to Equation (6) [41] (Figure 10 (green line)):

(6) $$T_{theoretical}=0.53I_{stack\;}+26.01$$

Additionally, as we have commented in Section 2.1.1, the stack includes a model NTC NB20N50104KBA temperature sensor, which, as we already mentioned, is the same model than the ones used in the implementation of the developed DAQ system.

Figure 10 (red line) shows the stack temperature variation with the load demand. The temperature data corresponds to those provided by the sensor with which the stack is equipped.

The results provided by the developed DAQ system are shown in Figure 11. This figure shows comparatively the 30 measuring points distributed over the entire surface of the stack. The temperature values read by the cell sensor included with the stack match the data line of the cell sensor #27-2 (black line).

**Figure 12:**
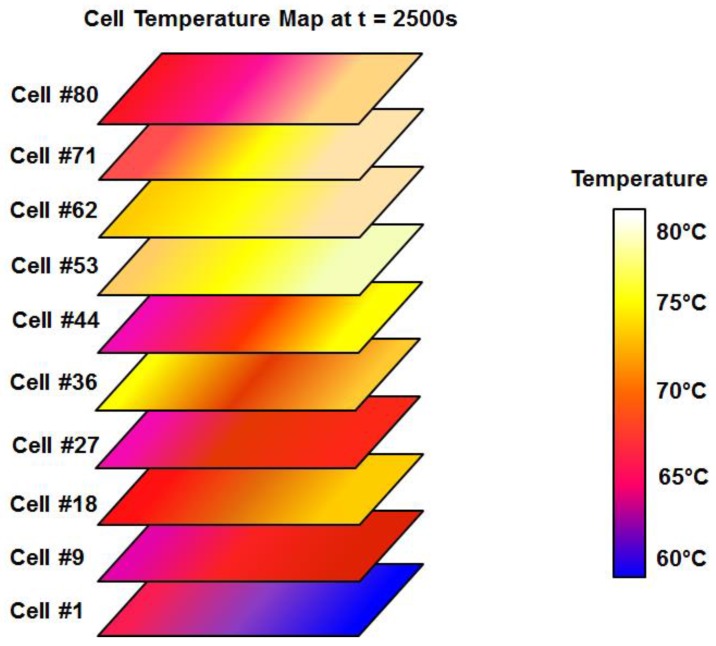
Cell temperature superficial map obtained from the developed DAQ system.

Once the temperature in the cells has been measured, it is possible to draw a surface temperature map like Figure 12. In this case, the data shown correspond to *t* = 2500 s, when the temperature in the cells achieves its highest value.

## 4. Discussion

In this work, a hardware/software DAQ system capable of monitoring in real time the cells temperature on an AC-PEFC has been presented. The importance of temperature in AC-PEFCs resides in the fact that fuel cell performance depends on the stack temperature. On the other hand, in AC-PEFCs, the stack temperature can vary according to the Oxidant/Cooling subsystem design. That is, the BoP configuration has an influence on the stack temperature and this in turn has an influence on the fuel cell performance.

Most manufacturers provide their commercial stacks equipped with at most one temperature sensor placed on a certain cell. Thus, a DAQ system for cell temperature monitoring will allow us to know the status of each cell. With this information, the user can prevent or avoid the failure of any particular cell.

Based on the experimental results, Figure 10 shows the stack temperature variation with the load demand, but only at one point in the stack (this point corresponds with the position of the sensor supplied by the manufacturer). The result indicates that the temperature at this point follows the expected (theoretical) behavior; however, this does not allow any inference as to whether the complete stack is functioning properly.

Moreover, of power loss in the stack due to a malfunction of cells by inadequate temperature (malfunction of the Oxidant/Cooling subsystem), or even deteriorated cells could occur. In this case, a temperature measurement of only one cell would not allow detection of the fault unless by chance the problem is circumscribed to the surroundings of the temperature sensor.

Paying attention now to Figure 11, which provides temperature data measured with the developed DAQ system, we can observe that the cells’ temperature follows the load profile in the same way as Figure 10. Nevertheless, the temperature rise time when the load current is going up is shorter than the fall time when the load current is going down. In this case, this is due to stack thermal capacity and to stack state (good or bad), not to a bad fan selection in the Oxidant/Cooling subsystem design. A detailed description of the BoP implementation can be found in [2], where it is demonstrated that the fan fulfils the oxidant/cooling airflow requirements for the entire operating range. Then, the temperature difference between the coldest and hottest point, reaching up to 20 °C at highest current point, provides us some clues about what cells are likely damaged.

Apart from this, we can observe the final temperature value achieved at the end of the test (30 °C), which corresponds with the initial value when the test started. This value coincides with the room temperature. Remember that it is an air-cooled PEFC stack, and the air is taken from the surroundings, so the temperature at rest state will depends on the environmental air temperature.

In case the test had been prolonged maintaining the stack at null load, but the fan blowing air and the room refreshed, the stack temperature would descend to the optimal temperature (26.01 °C) or even lower.

Additionally, from Figure 11 we can deduce that the coolest cells are those placed on the bottom side of the stack, cell #1–cell #9, while the hottest points are located around cell #53–cell #62. Moreover, paying attention to the sensor position, the cell sensor that corresponds with the position *T*_53-3_ (Figure 11- 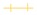 line, cell #53, right side) gives us the highest temperature value. This cell does not correspond to the cell that the manufacturer marks as the hottest point in the stack. As we have commented above, manufacturer indicates the middle of the stack (cell #40) as the hottest point, and estimates the highest temperature difference between the middle and the extremes at 8 °C. Therefore this contradiction with the manufacturer’s consideration leads us to think that cell #53 might be damaged.

Even more intuitively and at a glance, Figure 12 shows the effect mentioned in the previous paragraph. In it, we can observe which cells are worse cooled and even what side of each cell could be damaged. As it is easy to see, the sides of the edges and center of the cells are at different temperatures. Specifically, cell #53 has on its right side the highest temperature; conversely, cell #1 has the lowest temperature on its right side. Simply by analyzing the colors we can easily see that the difference between the hottest and coldest points on the stack is around 20 °C. Additionally, looking at any single cell, we can see temperature differences of about 5 °C between two points on the same surface. Therefore, the developed cell temperature monitoring DAQ system provides useful extra information complementary to that provided by the manufacturer based on a single temperature sensor.

The developed DAQ system allows knowledge of the stack temperature variations in two of its dimensions; height and length. All graphs analyzed together with the modular VI, provide very valuable information, both intuitive and accurate, about the behavior of the stack regarding the temperature of its cells. This allows preventive maintenance of the stack. It also permits the user to check whether the Oxidant/Cooling Subsystem is working properly. Moreover, it can be considered an excellent tool that helps to design an appropriate Oxidant/Cooling Subsystem, which is very necessary to develop a BoP for a given AC-PEFC stack. Finally, working together with the Instrumentation & Control subsystem, it allows optimization of the oxidant/cooling subsystem, redirecting the airflow in one way or another.

## 5. Conclusions

This work presents a hardware/software data acquisition system developed for monitoring the temperature of the cells in AC-PEFCs in real time. The system has been designed to be able to monitor each cell temperature along an AC-PEFC stack, save these data and process them to be able to carry out studies to know the degradation suffered by the cells, its origin and the way to extend the lifetime of the stack.

The main characteristics of the presented DAQ system for cell temperature monitoring that drive us to consider it a new contribution with respect existing commercial systems are its better resolution, lower number of wires, lower weight and the fact it does not need an additional power supply.

While an AC-PEFC is operating, the stack temperature changes over time and it depends on the load profile. The temperature has an influence on the electrochemical reaction rate and on water and reactant transport.

Most manufacturers supply their commercial stacks without any temperature sensor or at most only one sensor. With that, the user will have information about the operating temperature of only the one cell in which the sensor is placed. However, what about the rest of the cells in the stack?

Moreover, among the factors that affect the cell behavior in the stack, the cell temperature affects its ability to produce energy. Hence, the DAQ system for cell temperature monitoring is a critical concern in the PEFC scope, so the designed cell temperature acquisition and monitoring system tries to contribute to such a challenging issue.

Therefore, for these two purposes, a DAQ system for cell temperature monitoring has been developed and tested in this work. To build it, two widely used platforms like the Arduino Micro ATmega32u4, for hardware implementation, and NI LabVIEW, for software development, have been used. Additionally, the developed modular VI allows graphical and numerical visualizations configurable by the user. This gives to the user interface a great flexibility.

Experimental results show the hardware/software system works properly. It provides cell temperature measurements in three parts of the cell, allowing one to build a surface distribution temperature map along the stack.

The system has been conceived and carried out to fulfill the objectives of scalability, flexibility, easy-to-use, versatility and low cost. Moreover, according to the scientific literature, the authors have not found any similar cell temperature DAQ & monitoring systems with similar characteristics of scalability, flexibility, amount of information provided and its possibilities.

Furthermore, the developed DAQ system allows one to measure temperatures at the level of cell in stacks of any power (from watts to kilowatts). The stack power is related with the temperature gradient in the sense that for a higher power there will be a higher stack surface, and consequently higher temperature differences between the coldest and hottest point. In this practical case, a medium-sized power stack has been used for testing the developed DAQ system, and experimental tests have shown a difference around 20 °C between two extreme points of the stack.

We have now begun to integrate the presented DAQ system, as part of the Instrumentation & Control subsystem of the AC-PEFC test bench also developed by the authors of this work. Of course, the developed NI LabVIEW-based application will also be integrated into the SCADA that monitors and governs the AC-PEFC test bench.

## Figures and Tables

**Table 1:** DAQ system components characteristics.

NTC NB20N50104KBA	Arduino Micro Atmega32u4		MUX CD74HC4067	Op. Amp. LM358
*R*_0_ = 100 kΩ *B* = 4160 K *T_min_* = −55 °C and *T_max_* = 150 °C Tolerance = ± 10% Time constant = 7 s	*V_CC_* = 5 V I/O digital = 20 Analog inputs = 12 Clock = 16 MHz	DC current 40 mA Memory Flash 32 KB SRAM 2.5 KB EEPR OM 2.5 KB	*V_CC_* = 5 V *R_on_* = 65 Ω Analog inputs = 16	*V_CC_* = 5 V CMR = 70 dB Output voltage = 5 V

**Table 2:** Technical specifications of the developed DAQ system for cell temperature monitoring.

Tolerance = ± 10%	Size: 10 cm × 10 cm
Sample time: 400 ms	Auxiliary power supply NO
Weight: 50 g	Temperature range: (−55 °C to 150 °C)

## Abbreviations

The following abbreviations are used in this manuscript:

AC-PEFC	Air Cooled Polymer Electrolyte Fuel Cell
BoP	Balance of Plant
DAQ	Data Acquisition
FC	Fuel Cell
HT-FC	High Temperature Fuel Cell
LabVIEW	Laboratory Virtual Instrumentation Engineering Workbench
MUX	Multiplexer
NI	National Instrument
NTC	Negative Temperature Coefficient
Op. Amp.	Operational Amplifier
OS	Operating System
PE	Polymer Electrolyte
PEFC	Polymer Electrolyte Fuel Cell
PMS	Power Management System
PHM	Prognostics and Health Management
SCADA	Supervisory Control And Data Acquisition
VI	Virtual Instrument

## Appendix A

//////// TEMPERATURE MAP BASED ON TWO MUX //////////////////////////// //////////////////////////////////////////////TEP-192 ////////////////////////////////////////////////////////////////////////
//////////////////////////////////// CALIBRATED 2: Y=X+C ///////////////////////////////////////////////

#define NumCell 10
#define NumSensor 3

// MUX variables
#define S0 2
#define S1 3
#define S2 4
#define S3 5
#define E0 6
#define E1 7

// Variables CTN y voltaje divider

#define T0 298.15
#define Rfix 100000
#define Ro 100000
#define B 4160

#define temperature (B/(B/T0+log((Vctn*Rfix)/(Ro*(5-Vctn)))))-273

#define time 10 // iteration time

float T[NumCell][NumSensor];
float C[NumCell][NumSensor]; // CALIBRATE
float Taverage;
float Vctn;

char serie_rec;

// ---- LOOPS -----
int i,j;

void setup (){

Serial.begin(9600);

pinMode(S0,OUTPUT);
pinMode(S1,OUTPUT);
pinMode(S2,OUTPUT);
pinMode(S3,OUTPUT);
pinMode(E0,OUTPUT);
pinMode(E1,OUTPUT);

digitalWrite(S0,LOW);
digitalWrite(S1,LOW);
digitalWrite(S2,LOW);
digitalWrite(S3,LOW);
digitalWrite(E0,LOW);
digitalWrite(E1,HIGH);

for(i=0; i<NumCell; i++){
for (j=0; j<NumSensor; j++){
C[i][j]=1;
}
}
}

void loop(){

serie_rec = Serial.read();
if (serie_rec==‘A’) {FUNCALIBRATE(); serie_rec=0;}

// MUX ENABLE
digitalWrite(E1,HIGH); digitalWrite(E0,LOW);

// CELL 1
digitalWrite(S3,LOW); digitalWrite(S2,LOW); digitalWrite(S1,LOW); digitalWrite(S0,LOW); // 0000 0
delay(time); Vctn=(5.0/1024)*analogRead(0); T[0][0]=temperature; T[0][0]=T[0][0]+C[0][0];
digitalWrite(S3,LOW); digitalWrite(S2,LOW); digitalWrite(S1,LOW); digitalWrite(S0,HIGH); // 0001 1
delay(time); Vctn=(5.0/1024)*analogRead(0); T[0][1]=temperature; T[0][1]=T[0][1]+C[0][1];
digitalWrite(S3,LOW); digitalWrite(S2,LOW); digitalWrite(S1,HIGH); digitalWrite(S0,LOW); // 0010 2
delay(time); Vctn=(5.0/1024)*analogRead(0); T[0][2]=temperature; T[0][2]=T[0][2]+C[0][2];

// CELL 2
digitalWrite(S3,LOW); digitalWrite(S2,LOW); digitalWrite(S1,HIGH); digitalWrite(S0,HIGH); // 0011 3
delay(time); Vctn=(5.0/1024)*analogRead(0); T[1][0]=temperature; T[1][0]=T[1][0]+C[1][0];
digitalWrite(S3,LOW); digitalWrite(S2,HIGH); digitalWrite(S1,LOW); digitalWrite(S0,LOW); // 0100 4
delay(time); Vctn=(5.0/1024)*analogRead(0); T[1][1]=temperature; T[1][1]=T[1][1]+C[1][1];
digitalWrite(S3,LOW); digitalWrite(S2,HIGH); digitalWrite(S1,LOW); digitalWrite(S0,HIGH); // 0101 5
delay(time); Vctn=(5.0/1024)*analogRead(0); T[1][2]=temperature; T[1][2]=T[1][2]+C[1][2];

// CELL 3
digitalWrite(S3,LOW); digitalWrite(S2,HIGH); digitalWrite(S1,HIGH); digitalWrite(S0,LOW); // 0110 6
delay(time); Vctn=(5.0/1024)*analogRead(0); T[2][0]= temperature; T[2][0]=T[2][0]+C[2][0];
digitalWrite(S3,LOW); digitalWrite(S2,HIGH); digitalWrite(S1,HIGH); digitalWrite(S0,HIGH); // 0111 7
delay(time); Vctn=(5.0/1024)*analogRead(0); T[2][1]= temperature; T[2][1]=T[2][1]+C[2][1];
digitalWrite(S3,HIGH); digitalWrite(S2,LOW); digitalWrite(S1,LOW); digitalWrite(S0,LOW); // 1000 8
delay(time); Vctn=(5.0/1024)*analogRead(0); T[2][2]= temperature; T[2][2]=T[2][2]+C[2][2];

// CELL 4
digitalWrite(S3,HIGH); digitalWrite(S2,LOW); digitalWrite(S1,LOW); digitalWrite(S0,HIGH); // 1001 9
delay(time); Vctn=(5.0/1024)*analogRead(0); T[3][0]= temperature; T[3][0]=T[3][0]+C[3][0];
digitalWrite(S3,HIGH); digitalWrite(S2,LOW); digitalWrite(S1,HIGH); digitalWrite(S0,LOW); // 1010 10
delay(time); Vctn=(5.0/1024)*analogRead(0); T[3][1]= temperature; T[3][1]=T[3][1]+C[3][1];
digitalWrite(S3,HIGH); digitalWrite(S2,LOW); digitalWrite(S1,HIGH); digitalWrite(S0,HIGH); // 1011 11
delay(time); Vctn=(5.0/1024)*analogRead(0); T[3][2]= temperature; T[3][2]=T[3][2]+C[3][2];

// CELL 5
digitalWrite(S3,HIGH); digitalWrite(S2,HIGH); digitalWrite(S1,LOW); digitalWrite(S0,LOW); // 1100 12
delay(time); Vctn=(5.0/1024)*analogRead(0); T[4][0]= temperature; T[4][0]=T[4][0]+C[4][0];
digitalWrite(S3,HIGH); digitalWrite(S2,HIGH); digitalWrite(S1,LOW); digitalWrite(S0,HIGH); // 1101 13
delay(time); Vctn=(5.0/1024)*analogRead(0); T[4][1]= temperature; T[4][1]=T[4][1]+C[4][1];
digitalWrite(S3,HIGH); digitalWrite(S2,HIGH); digitalWrite(S1,HIGH); digitalWrite(S0,LOW); // 1110 14
delay(time); Vctn=(5.0/1024)*analogRead(0); T[4][2]= temperature; T[4][2]=T[4][2]+C[4][2];

// CELL 6
digitalWrite(S3,HIGH); digitalWrite(S2,HIGH); digitalWrite(S1,HIGH); digitalWrite(S0,HIGH); // 1111 15
delay(time); Vctn=(5.0/1024)*analogRead(0); T[5][0]=temperature; T[5][0]=T[5][0]+C[5][0];

// MUX ENABLE
digitalWrite(E0,HIGH); digitalWrite(E1,LOW);

digitalWrite(S3,LOW); digitalWrite(S2,LOW); digitalWrite(S1,LOW); digitalWrite(S0,LOW); // 0000 0
delay(time); Vctn=(5.0/1024)*analogRead(0); T[5][1]=temperature; T[5][1]=T[5][1]+C[5][1];
digitalWrite(S3,LOW); digitalWrite(S2,LOW); digitalWrite(S1,LOW); digitalWrite(S0,HIGH); // 0001 1
delay(time); Vctn=(5.0/1024)*analogRead(0); T[5][2]=temperature; T[5][2]=T[5][2]+C[5][2];

// CELL 7
digitalWrite(S3,LOW); digitalWrite(S2,LOW); digitalWrite(S1,HIGH); digitalWrite(S0,LOW); // 0010 2
delay(time); Vctn=(5.0/1024)*analogRead(0); T[6][0]= temperature; T[6][0]=T[6][0]+C[6][0];
digitalWrite(S3,LOW); digitalWrite(S2,LOW); digitalWrite(S1,HIGH); digitalWrite(S0,HIGH); // 0011 3
delay(time); Vctn=(5.0/1024)*analogRead(0); T[6][1]= temperature; T[6][1]=T[6][1]+C[6][1];
digitalWrite(S3,LOW); digitalWrite(S2,HIGH); digitalWrite(S1,LOW); digitalWrite(S0,LOW); // 0100 4
delay(time); Vctn=(5.0/1024)*analogRead(0); T[6][2]= temperature; T[6][2]=T[6][2]+C[6][2];

// CELL 8
digitalWrite(S3,LOW); digitalWrite(S2,HIGH); digitalWrite(S1,LOW); digitalWrite(S0,HIGH); // 0101 5
delay(time); Vctn=(5.0/1024)*analogRead(0); T[7][0]=temperature; T[7][0]=T[7][0]+C[7][0];
digitalWrite(S3,LOW); digitalWrite(S2,HIGH); digitalWrite(S1,HIGH); digitalWrite(S0,LOW); // 0110 6
delay(time); Vctn=(5.0/1024)*analogRead(0); T[7][1]= temperature; T[7][1]=T[7][1]+C[7][1];
digitalWrite(S3,LOW); digitalWrite(S2,HIGH); digitalWrite(S1,HIGH); digitalWrite(S0,HIGH); // 0111 7
delay(time); Vctn=(5.0/1024)*analogRead(0); T[7][2]= temperature; T[7][2]=T[7][2]+C[7][2];

// CELL 9
digitalWrite(S3,HIGH); digitalWrite(S2,LOW); digitalWrite(S1,LOW); digitalWrite(S0,LOW); // 1000 8
delay(time); Vctn=(5.0/1024)*analogRead(0); T[8][0]=temperatura; T[8][0]=T[8][0]+C[8][0];
digitalWrite(S3,HIGH); digitalWrite(S2,LOW); digitalWrite(S1,LOW); digitalWrite(S0,HIGH); // 1001 9
delay(time); Vctn=(5.0/1024)*analogRead(0); T[8][1]=temperatura; T[8][1]=T[8][1]+C[8][1];
digitalWrite(S3,HIGH); digitalWrite(S2,LOW); digitalWrite(S1,HIGH); digitalWrite(S0,LOW); // 1010 10
delay(time); Vctn=(5.0/1024)*analogRead(0); T[8][2]=temperatura; T[8][2]=T[8][2]+C[8][2];

// CELL 10
digitalWrite(S3,HIGH); digitalWrite(S2,LOW); digitalWrite(S1,HIGH); digitalWrite(S0,HIGH); // 1011 11
delay(time); Vctn=(5.0/1024)*analogRead(0); T[9][0]=temperature; T[9][0]=T[9][0]+C[9][0];
digitalWrite(S3,HIGH); digitalWrite(S2,HIGH); digitalWrite(S1,LOW); digitalWrite(S0,LOW); // 1100 12
delay(time); Vctn=(5.0/1024)*analogRead(0); T[9][1]=temperature; T[9][1]=T[9][1]+C[9][1];
digitalWrite(S3,HIGH); digitalWrite(S2,HIGH); digitalWrite(S1,LOW); digitalWrite(S0,HIGH); // 1101 13
delay(time); Vctn=(5.0/1024)*analogRead(0); T[9][2]=temperature; T[9][2]=T[9][2]+C[9][2];

Serial.print (“A”); Serial.print(T[0][0]);
Serial.print (“B”); Serial.print(T[0][1]);
Serial.print (“C”); Serial.print(T[0][2]);

Serial.print (“D”); Serial.print(T[1][0]);
Serial.print (“E”); Serial.print(T[1][1]);
Serial.print (“F”); Serial.print(T[1][2]);

Serial.print (“G”); Serial.print(T[2][0]);
Serial.print (“H”); Serial.print(T[2][1]);
Serial.print (“I”); Serial.print(T[2][2]);

Serial.print (“J”); Serial.print(T[3][0]);
Serial.print (“K”); Serial.print(T[3][1]);
Serial.print (“L”); Serial.print(T[3][2]);

Serial.print (“M”); Serial.print(T[4][0]);
Serial.print (“N”); Serial.print(T[4][1]);
Serial.print (“O”); Serial.print(T[4][2]);

Serial.print (“P”); Serial.print(T[5][0]);
Serial.print (“Q”); Serial.print(T[5][1]);
Serial.print (“R”); Serial.print(T[5][2]);

Serial.print (“S”); Serial.print(T[6][0]);
Serial.print (“T”); Serial.print(T[6][1]);
Serial.print (“U”); Serial.print(T[6][2]);

Serial.print (“V”); Serial.print(T[7][0]);
Serial.print (“W”); Serial.print(T[7][1]);
Serial.print (“X”); Serial.print(T[7][2]);

Serial.print (“Y”); Serial.print(T[8][0]);
Serial.print (“Z”); Serial.print(T[8][1]);
Serial.print (“a”); Serial.print(T[8][2]);

Serial.print (“b”); Serial.print(T[9][0]);
Serial.print (“c”); Serial.print(T[9][1]);
Serial.print (“d”); Serial.print(T[9][2]);
Serial.print (“e”);
}

void FUNCALIBRATE(){
// Yi=Ci+xi

// Cálculo de Tmedia
Tmedia=0;
for(i=0; i<NumsCell; i++){
for (j=0; j<NumSensor; j++){
Taverage=Taverage+T[i][j];
}
}
Taverage=Taverage/30;
// Cálculo de Constante C
for(i=0; i<NumCell; i++){
for (j=0; j<NumSensor; j++){
C[i][j]= Taverage-T[i][j];
}
}
}
